# A protocol for the use of cloud-based quantum computers for logical network analysis of biological systems

**DOI:** 10.1016/j.xpro.2023.102438

**Published:** 2023-08-06

**Authors:** Felix M. Weidner, Mirko Rossini, Joachim Ankerhold, Hans A. Kestler

**Affiliations:** 1 Institute of Medical Systems Biology, University of Ulm, 89081 Ulm, Germany; 2 Institute of Complex Quantum Systems, University of Ulm, 89081 Ulm, Germany; 3 Integrated Quantum Science and Technology (IQST), Ulm University & Stuttgart University & Max Planck Institute for Solid State Research, Ulm, Germany; 4 Leibniz Institute on Aging - Fritz Lipmann Institute (FLI), 07745 Jena, Germany

**Keywords:** Systems biology, Computer sciences

## Abstract

Boolean networks are commonly used in systems biology to dynamically model gene regulatory interactions. Here, we present a protocol for implementing Boolean network dynamics as quantum circuits. We describe steps for accessing cloud-based quantum processing units offered by IBM and IonQ and downloading and parsing logic for gene regulatory networks. We then detail procedures for performing simulations of quantum circuits on local devices and visualizing measurement results.

For complete details on the use and execution of this protocol, please refer to Weidner et al.^1^

## Highlights

• Downloading and parsing logic for gene regulatory networks
• Setting up cloud accounts to access real quantum processing units
• Running local simulations of quantum circuits to analyze network dynamics
• Transpiling circuits to be run on real quantum computers and sending out tasks

## Abstract (editor highlights)

Publisher’s note: Undertaking any experimental protocol requires adherence to local institutional guidelines for laboratory safety and ethics.

## Graphical abstract

**Figure:**
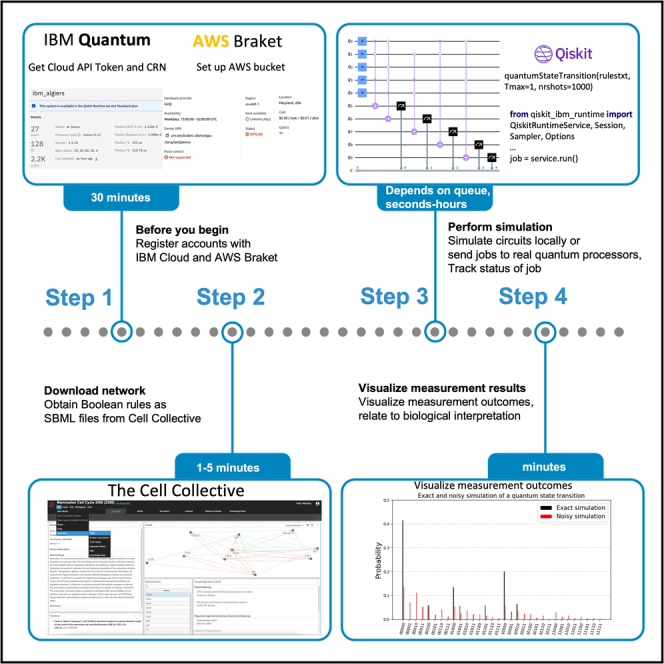

## Before you begin

Before describing the creation of quantum circuits for analyses of logical networks, we show the account setup process for obtaining access to cloud-based quantum processors.

### Setting up an account for use of the IBM quantum cloud

**Timing: 30–45 min**

In this section, we set up all the needed requirements to run jobs on the IBM quantum devices through the IBM Quantum Cloud platform.

***Note:*** IBM has introduced a pay-as-you-go scheme, which allows access to 27-qubit quantum processors without requiring membership to the IBM Quantum network. Prices are currently at 1.60$ per second of runtime on the device, payable via credit card or IBM Cloud credits.

In the following we explain how to set up an account and the required instance to send tasks to these devices.

1. Go to the registration portal at https://cloud.ibm.com/registration?target=%2Fquantum.
2. Follow the steps the portal requires:
  a. Provide a valid e-mail and password for the new account and verify the account.
  b. Provide required information regarding billing details, then confirm the provided bank account by accepting a billing request for 0,00$ which serves to check the validity of the provided credit card.

***Note:*** At the time of publication, IBM is offering a free trial of up to $200 worth of simulation time on the platform, valid for 35 days. However, this is listed as a special limited time offer.

**Figure 1:**
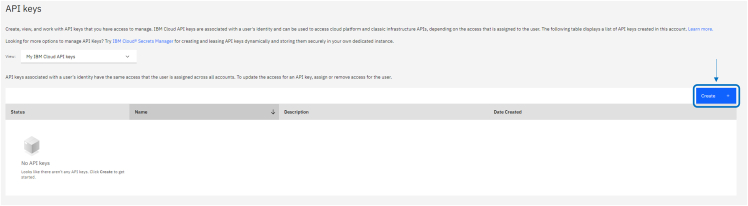
Snapshot of the API key interface of the IBM Quantum cloud
  b. Add a name and a description for the API key to be created, then press on the blue button “Create”.
    ***Note:*** This key uniquely identifies a user and will be required later in step 5a. For further documentation on API keys for IBM Cloud services, please check at https://www.ibm.com/docs/en/app-connect/container?topic=servers-creating-cloud-api-key.
  c. The API key can be retrieved from the page that opens right after the one shown in Figure 2, as shown in Figure 3:
    ***Optional:*** Here one can visualize the API key by pressing on the eye-shaped icon, then copy or download it by using the two buttons on the lower right corner. The downloaded file is a .json formatted text which can be opened by a text editor as shown in Figure 4
    **CRITICAL:** It will not be possible to retrieve this code from the website, so it is crucial to save it in a secure place! Furthermore, it is strongly suggested to keep this identifying code private, as they allow for interaction with IBM services. The posting of this information on open-source platforms such as GitHub repositories could be an opportunity for account theft.

3. Once logged in, go to the webpage https://cloud.ibm.com/iam/apikeys. There, to create the API key needed to log in to the IBM services through Qiskit, follow these steps:
  a. Press “Create” on the blue button at top right of the table as in Figure 1.

**Figure 2:**
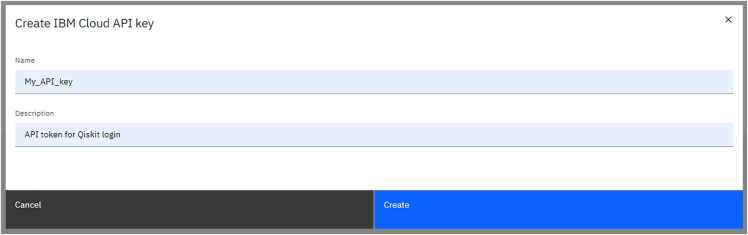
Snapshot of the API key creation interface of the IBM Quantum cloud

**Figure 3:**
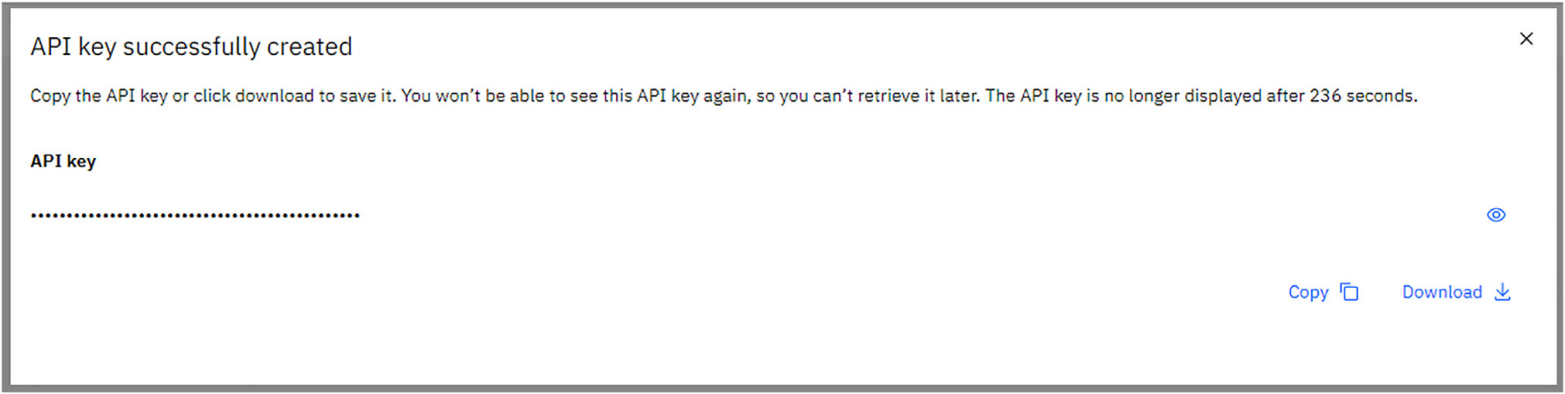
Snapshot of the interface needed to retrieve the API key on the IBM Quantum cloud

**Figure 4:**
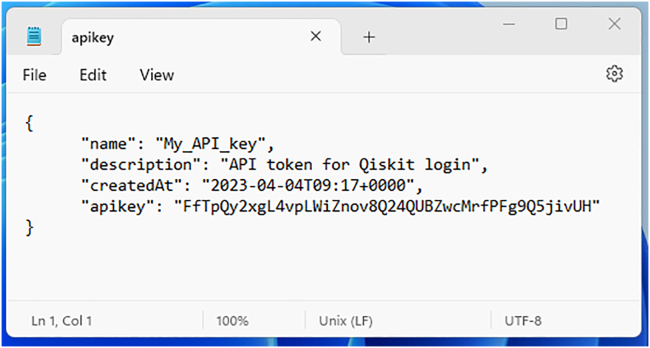
Snapshot of the .json file containing the API key, opened on the Windows text reader
4. The next step is to create an instance and retrieve from there the required CRN (Cloud Resource Name) for the login. To create the CRN:
  a. Go to the following webpage: https://cloud.ibm.com/quantum/instances and press on the “Create instance” blue button. This redirects you to a different webpage.
  b. In the new page, select the price plan required. The Lite plan, with free access, allows us to access only to the quantum device simulators. To access to the real devices, select the Standard plan (see red box in Figure 5).

**Figure 5:**
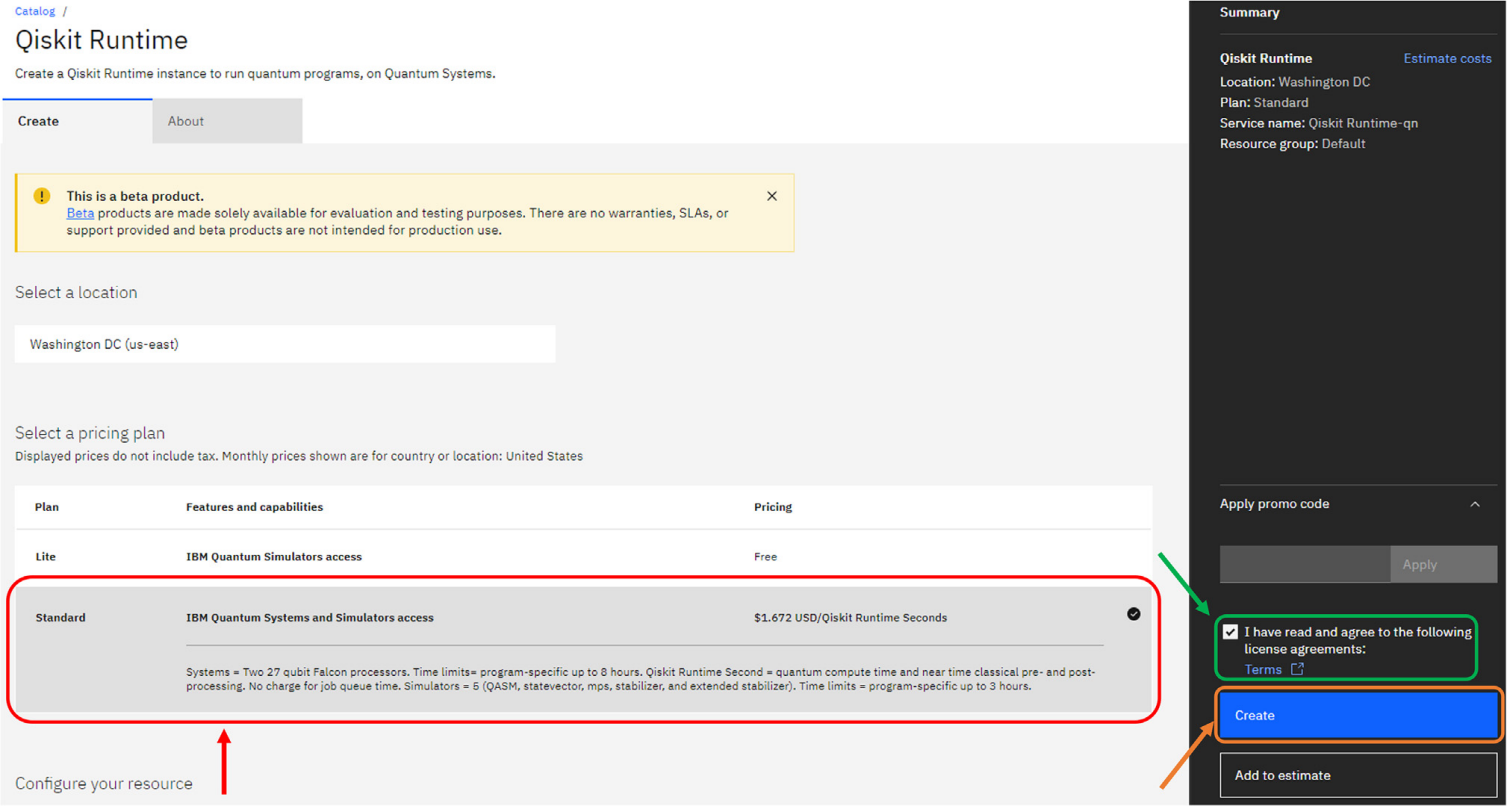
Snapshot of the webpage dedicated to the creation of an instance on the IBM Quantum Cloud
  c. Check on the green highlighted box to accept license agreements.
  d. Click on the blue “Create” button.
  e. In the following page you can retrieve your CRN as shown in Figure 6, highlighted in green. This code, together with the API key retrieved before, will be used in step 5a.

**Figure 6:**
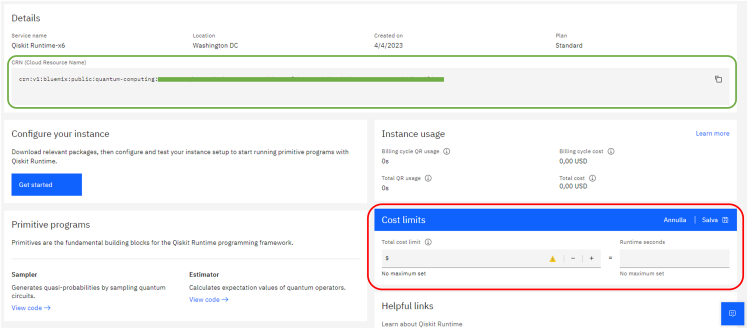
Snapshot of the webpage where the CRN code can be retrieved, in the green box

***Note:*** This code serves as a globally unique identifiers for cloud resources. For further documentation on the role of CRNs for IBM Cloud computing, please check https://cloud.ibm.com/docs/account?topic=account-crn

**CRITICAL:** The red-highlighted box in Figure 6 shows where to set a time limit for the computation run on the quantum device. Remember to set a time limit according to your needs to avoid unnecessary costs due to coding or algorithmic errors!

5. Now we connect to the IBM instance we just created from our notebook (STARProtocol.ipynb), accessing the computational resources we need. To do so:
  a. Save on disk the account credentials using the following code (you need to insert in quotation marks the API key from step 3c and the CRN from step 4e):
    apikey = "**ENTER-YOUR-IBMCLOUD-API-KEY**"
    crn = "**ENTER-YOUR-INSTANCES-CLOUD-RESOURCE-NUMBER**"
    name = "**StarProtocol**"
    # *Save an IBM Cloud account on disk and give it a name*.
    QiskitRuntimeService.save_account(channel="**ibm_cloud**", token=apikey, instance=crn, name=name)
  b. Connect to the service by calling the account as follows:

service = QiskitRuntimeService(name="**StarProtocol**")

Everything is now in place to run the necessary algorithms on a simulator or a real quantum device.

### Setting up an account for use of the IonQ QPU with Amazon Braket

**Timing: 45–60 min**

In this section we set up all the needed requirements to run jobs on the IonQ quantum device through the Amazon Braket platform.

***Note:*** The first quantum processor made publicly accessible by the company IonQ consists of 11 qubits based on trapped ions, and is called ‘IonQ Harmony’. This device can be accessed via multiple options, including Amazon Braket, Microsoft Azure, Google Cloud as well as IonQ’s own Quantum Cloud (see https://ionq.com/quantum-cloud#access-options).

For further details about this quantum processor, the technology enabling its features and more, an overview is available at https://ionq.com/quantum-systems/harmony.

Currently, the cost of using this processor consists of a flat 0.30$ USD per task plus 0.01$ USD for each shot (i.e., for each run of a quantum circuit followed by a measurement).

Here, we will focus on access via Amazon Braket and its associated python SDK.

**Figure 7:**
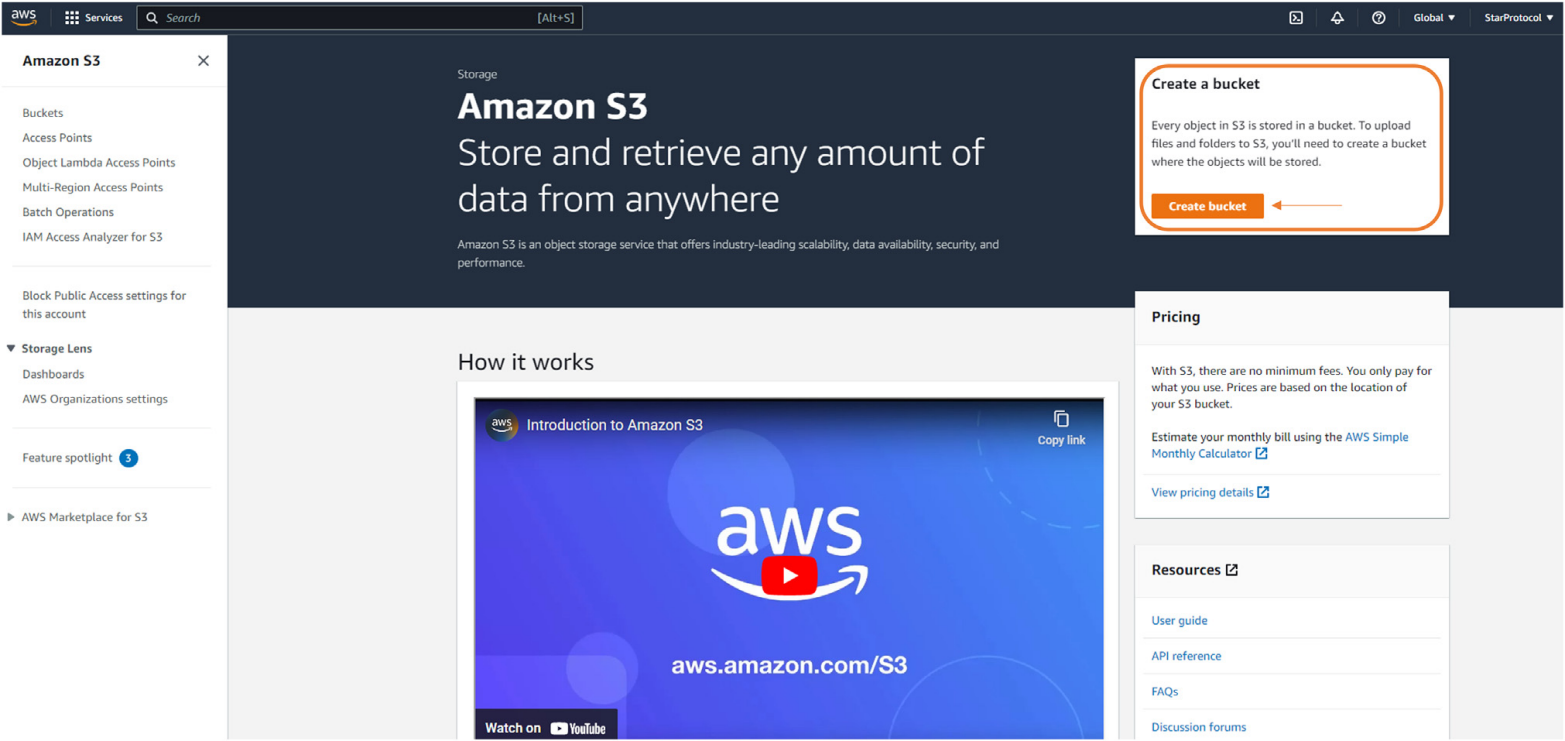
Snapshot of the Amazon Braket webpage for initiating the creation of a bucket
8. In the next page insert a name for the bucket on creation and follow the steps to complete the settings of the bucket. The name of the bucket should begin with “amazon-braket-“ followed by the desired name.

***Note:*** Default settings work for the purpose of this protocol.

**Figure 8:**
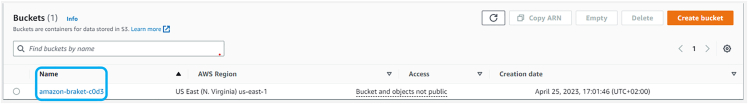
Snapshot of the Amazon Braket bucket interface

***Note:*** This page lists all the existing buckets, including the one just created.

6. Sign up to the AWS platform:
  a. Go to https://portal.aws.amazon.com/billing/signup#/start/email;
  b. Insert a valid e-mail and account name, the e-mail will be verified through verification code. Afterward, set a password.
  c. Insert all required data about account holder and bank account, then verify your bank account and identity by following the provided instructions.
  d. Select basic support in the following page to complete the account setup.
  e. Log into your account using the provided email and password.
7. Once logged in, go to the page https://us-east-1.console.aws.amazon.com/s3/get-started?region=us-east-1 and click on “Create bucket” as shown in Figure 7.

9. In the following page, click on the name of the bucket to enter its setting page, as shown in Figure 8.

**CRITICAL:** The name of the bucket, under “Name”, will be important as a parameter to insert in the working notebook.

**Figure 9:**
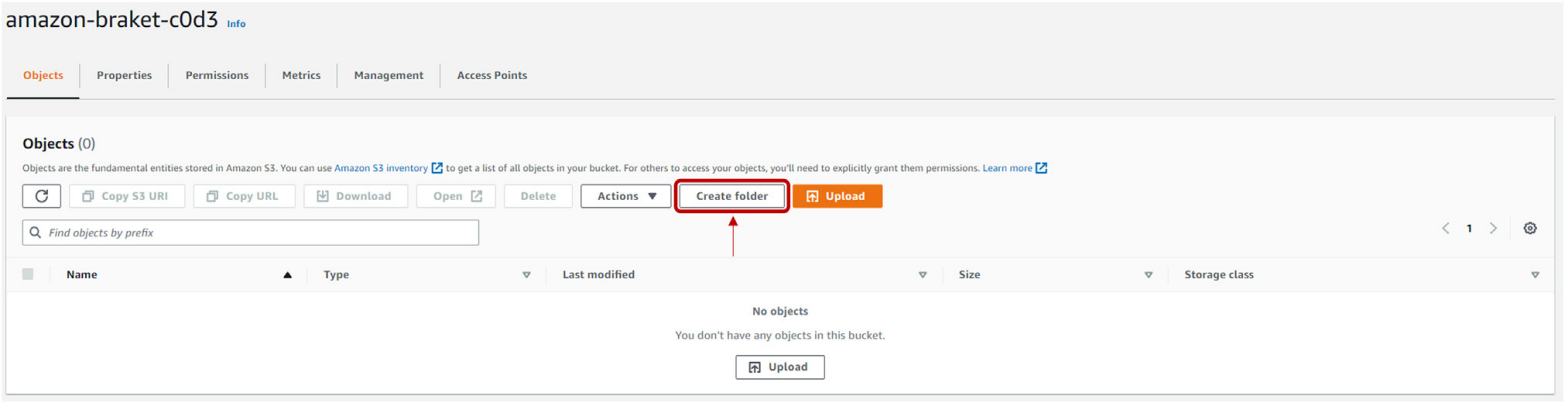
Snapshot of the Amazon Braket bucket settings interface

***Note:*** Here, all the results of the quantum computation on the IonQ device will be stored.

10. In the following page click on “Create folder” as shown in Figure 9 and, in the interface opening this way, insert a valid name for the created folder.

11. After creating the folder, the page is redirected to the page shown in Figure 8. Save the name of this folder as you will need later to insert it in the notebook.

***Note:*** Now we retrieve the access key and secret access key: these will be used for a one-time initial setup of the AWS services on a local device. To do so:

12. Go to https://console.aws.amazon.com/iam/
13. Click on “Users”, as shown in Figure 10.

**Figure 10:**
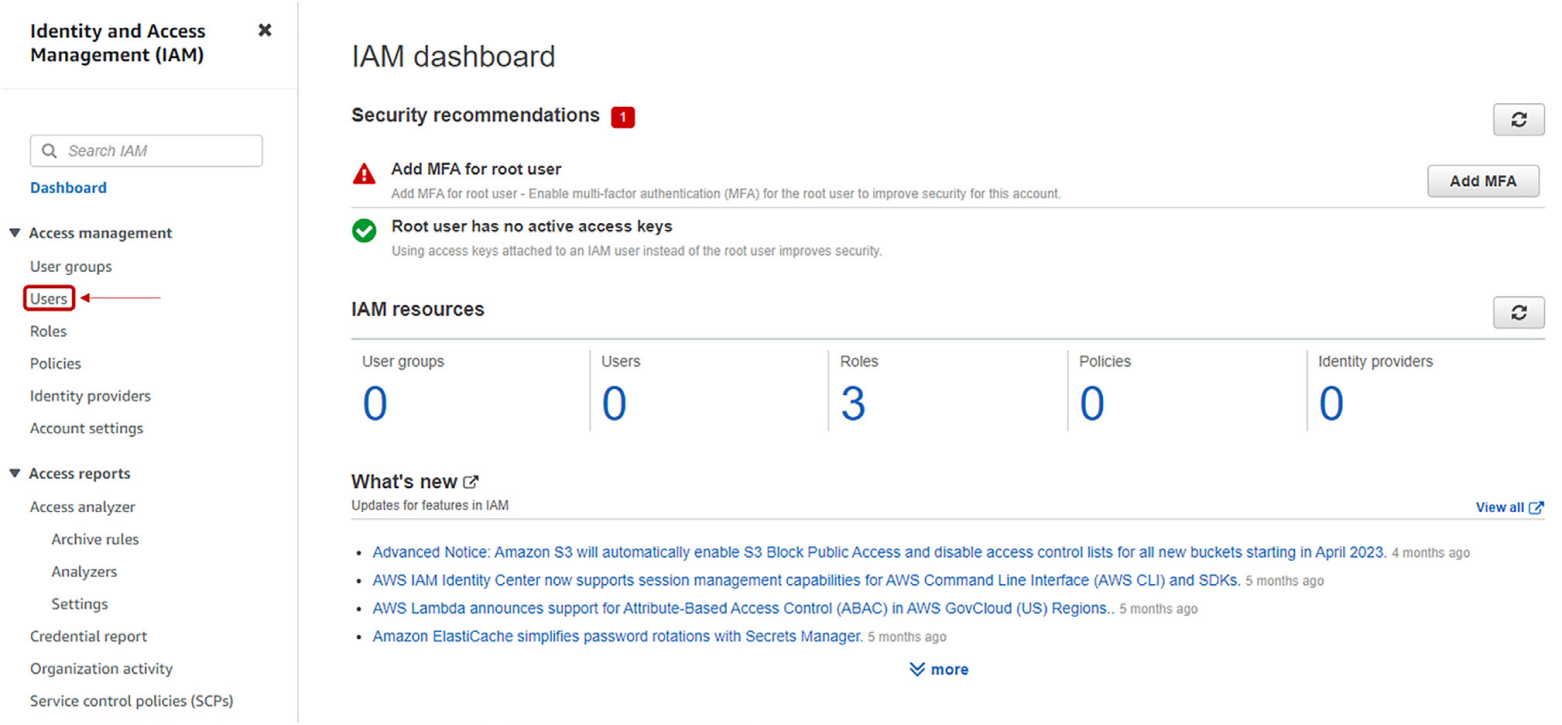
IAM dashboard snapshot, highlighted the button to move to User's interface
14. In the new window, click on “Add users”, if a user is not already present, as shown in Figure 11.

**Figure 11:**
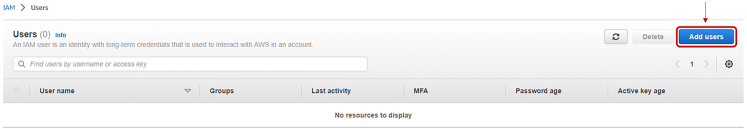
User interface snapshot, highlighted the button to create a new User
15. In the next page, insert a valid username in the dedicated box and press “next”, then keep the default options and press again “next”. Finally, on the next window press “Create user”.
16. The webpage now redirects on the one shown in Figure 11, where now appears the newly created user. Access the settings of the user by clicking on the username.
17. In the newly opened page, as shown in Figure 12, click firstly on “Security credentials” and then on “Create access key”.

**Figure 12:**
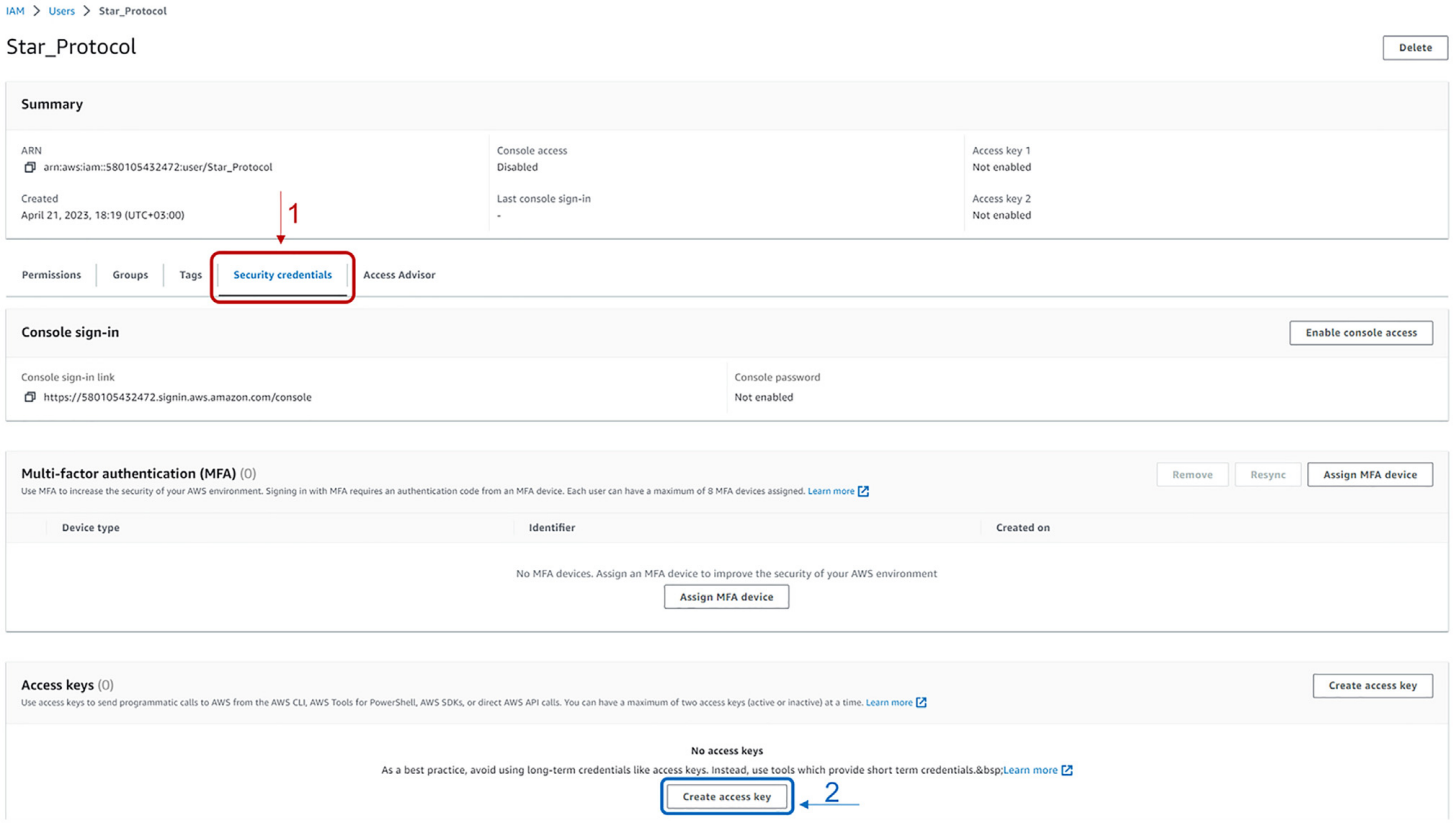
Snapshot of the panel leading to the creation of Access Key and Secret Access key
18. In the next window, select “Other” from the dotted list and press “Next”. Then, in the following page, optionally insert a tag name for the access key and press “Create access key”.
19. Now it is possible to retrieve the Access key and the Secret access key.

**Figure 13:**
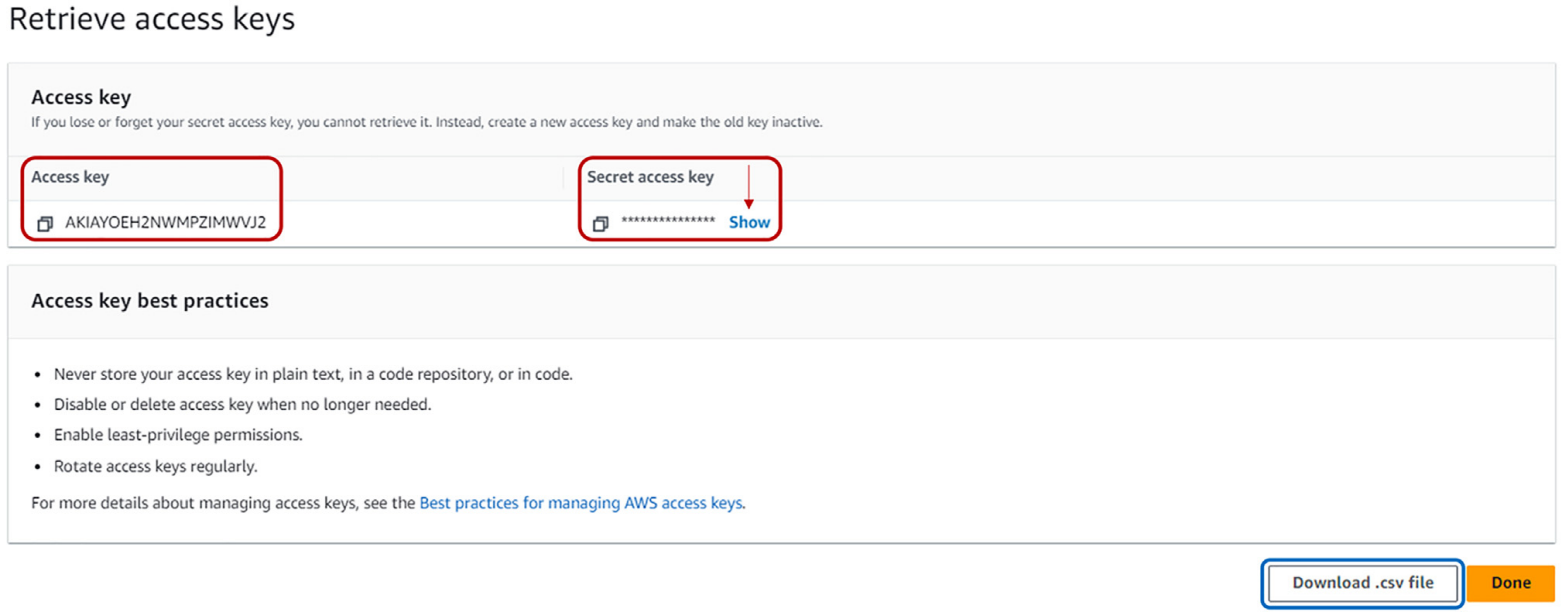
Snapshot of the panel containing Access Key and Secret Access Key

**CRITICAL:** the latter can only be retrieved from this window and will not be shown again! By pressing on “**Show**” it is possible to see and copy the Secret access key. By clicking on “Download .csv file” it is possible to download these two keys in a .csv file readable by any text editor. This is shown in Figure 13.

***Note:*** The next step requires the AWS CLI. It can be downloaded for all major operating systems from https://docs.aws.amazon.com/cli/latest/userguide/getting-started-install.html#getting-started-install-instructions. Once on the website, follow the instructions to download and install it. To check whether the installation was successful, open your terminal or PowerShell and run the command

> aws --version

20. Finally, these keys must be stored locally on the device. To save the keys, execute the following command

> aws configure

and follow the prompted instructions. When asked for the region name, provide the one listed in the bucket, as seen in Figure 8.

## Key resources table

**Table:** 

REAGENT or RESOURCE	SOURCE	IDENTIFIER
**Software and algorithms**		

R	R Core Team^2^	https://r-project.org/
BoolNet	Müssel et al.^3^	https://CRAN.R-project.org/package=BoolNet
Python	Van Rossum et al.^4^	https://github.com/python
Qiskit	Cross^5^	https://github.com/Qiskit
Qiskit Runtime	Johnson^6^	https://github.com/Qiskit/qiskit-ibm-runtime
AWS Braket	Gonzalez^7^	https://github.com/aws/amazon-braket-sdk-python

**Other**		

Boolean networks	Schwab et al.^8^	https://doi.org/10.1016/j.csbj.2020.03.001. eCollection 2020.
Cortical Area Network	Giacomantonio and Goodhill^9^	https://github.com/sysbio-bioinf/QuantumSTARProtocol, Helikar et al.^10^
Cell Cycle Network	Fauré et al.^8^	https://github.com/sysbio-bioinf/QuantumSTARProtocol, Helikar et al.^10^
IBM Quantum processors	IBM	https://cloud.ibm.com/login?redirect=%2Fquantum
IonQ ‘Harmony‘ quantum processor	IonQ	https://ionq.com/quantum-systems/harmony

## Step-by-step method details

As the account setups are now complete, you can begin with the analysis of Boolean networks.

### Obtaining network rules from Cell Collective in SBML format

**Timing: 5 min**

In this section we show how to download the required Boolean networks from the Cell Collective database.

1. Go to https://cellcollective.org/^10^
2. Click the ‘Get Started as Researcher’ button.
3. Find a Boolean network to analyze. The  icon on the top right can be used show the available models in a list. Boolean networks are indicated by the ‘boolean’ value in the ‘Type’ column.

***Note:*** In this protocol, we will focus on the networks of Giacomantonio and Goodhill^9^ and Fauré et al.^11^ These models are available under the names “Cortical Area Development” and “Mammalian Cell Cycle 2006” respectively.

4. Click ‘File’ --> ‘Download’ --> ‘SBML’ to obtain the Boolean rules of the model as a .sbml file. This format can be further processed using the BoolNet R-package.^3^

**Alternative**: Use the Jupyter notebook “STARProtocol.ipynb” along with the two networks provided as txt files in the ‘networks’ folder of the GitHub repository associated with this protocol (https://github.com/sysbio-bioinf/QuantumSTARProtocol). The files for any other Boolean network to be analyzed should be placed in the same ‘networks’ folder.

### Installing BoolNet to convert network rules into txt format

**Timing: 5 min**

We here explain how to use the BoolNet package for R for parsing the networks obtained in the previous section.

5. Download and install the latest version of R following the instructions at https://cran.r-project.org/ for your operating systems.
6. Download and install RStudio following the instructions at https://rstudio.com/ide for your operating system.
7. Open RStudio and run the command

> install.packages("BoolNet")

in the console to install the latest version of BoolNet^3^ from CRAN.

8. Run the function convertRules() in the file convertRules.R provided on the GitHub repository. This takes the arguments ‘SBML’ and ‘savePath’ for the paths where the provided SBML file is located as well as the desired location to save the .txt file.

***Note:*** Boolean networks may contain time delays, meaning that the update of a gene’s state relies on that gene’s state more than one time step in the past. When networks are obtained from the Cell Collective and represented using BoolNet notation in txt format, such delays are indicated by square brackets. E.g. the appearance of a term such as “Fgf8[-2]” in a Boolean rule means that the rule’s output is calculated based on the value of Fgf8 two time steps in the past. The proposed methods can not be applied to networks containing such time delays in their rules.

### Installing software packages to run algorithms on quantum devices

**Timing: 5 min**

In this section, we explain an alternative way to install the required Python packages, including the Qiskit environment, which does not rely on the provided Jupyter notebook directly.

In the specified GitHub repository, we provide a requirements.txt file which can be used to install all libraries that are required for the following analyses. This includes, among others, the Qiskit and Amazon Braket SDKs for the creation and simulation of quantum circuits, and the qiskit-ibm-runtime library for sending tasks to real IBM quantum processors using the Runtime environment.

To do so:

9. Create a new virtual environment in the programming language Python named ‘myenv’ using the command

$ python -m venv myenv

or.

$ conda create --name myenv

if Conda is installed.

10. Activate this environment using

$ source myenv/bin/activate

11. Install the libraries locally using the command

$ pip install -r requirements.txt

with the downloaded file.

### Mapping Boolean rules to quantum circuits

**Timing: seconds**

In this section we describe how the Boolean logic can be mapped into quantum circuits. This process is an essential block for the algorithms described further below.

The function *parse_BoolNetRules(rulestxt = ”./networks/Giacomantonio2010.txt”, saveDir = None)* provided in the *functionsForProtocol.py* file takes the argument ‘rulestxt’, which specifies the path to the text file containing the Boolean network rules (e.g., for the network of Giacomantonio), and returns a Python file containing the instructions to build the associated quantum circuit.

This is done by.

12. Import the file “functionsForProtocol.py” in the Jupyter notebook.
13. Run the parse_BoolNetRules() function. This generates a Python file with the name of the network, with the ending “_rulefile.py”.

***Note:*** The generated “_rulefile.py” file includes all Boolean rules used to synthesize quantum circuits for performing state transitions. These are defined using ‘@classical_function’ from the qiskit.circuit library. The package ‘tweedledum v1.1.1’ was used for the parsing of Boolean logic into quantum circuits.

### Simulating the dynamics of a quantum Boolean network locally

**Timing: seconds**

In this section we simulate the dynamics of Boolean networks on quantum devices using the local processor. This procedure is commonly used for testing the created quantum circuit before sending it to a real quantum device.

***Note:*** The main function used for generating circuits performing state transitions is *quantumStateTransition()*. This function generates circuits of (T+1)n qubits to simulate T state transitions of an n component network, with each time step being mapped to a separate set of qubits. Qubits are initially in the ‘0’ state by default. The first set of n qubits of these circuits is initialized with a layer of Hadamard gates, creating a uniformly weighted superposition of all $N=2^{n}$ states. All dynamical analyses are presented using the n = 5 cortical area development network of Giacomantonio and Goodhill^9^ as an example, as shown in the Jupyter notebook.

14. Call the function as shown in the following code snippet, which generates a quantum circuit for performing T=4 consecutive quantum state transitions from an initial uniform superposition state and measures the final register of n qubits, which will stochastically collapse the superposition state on one of the network’s two attractors.

***Note:*** This process of initializing, running and measuring the circuit is called a shot, which is then repeated 1000 times, yielding a probability distribution of measurement outcomes.

*#Functions and network file required for all further analyses*

**from** functionsForProtocol **import** ∗

rulestxt = "**./networks/Giacomantonio2010.txt**"

CorticalNet4Transitions = quantumStateTransition(rulestxt, Tmax=4, nrshots=1000)

print(CorticalNet4Transitions)

***Note:*** Given the order of components specified in *Giacomantonio2010.txt* this returns the unperturbed classical Boolean network’s attractor distribution of the states 10010 and 01101 with probabilities of 87.5% and 12.5% respectively. These probabilities are equivalent to the attractor’s basin sizes, which denote the relative number of states flowing into a given attractor. In Qiskit, bitstrings denoting gene activity are read from right to left.

### Adding noise profiles to a simulator mimicking a real IBM quantum processor

**Timing: seconds to minutes**

In this section we simulate a single noisy state transition in the cortical area network using a fake backend imitating the noise profile of a real IBM quantum device.

***Note:*** In addition to exact, noiseless simulations, Qiskit offers the possibility of testing the results and performance of a quantum circuit on simulators that can mimic the error rates of the various real quantum processors by IBM. These are referred to as ‘fake’ or ‘mock’ backends. These backends do not access a real device and thus do not require the creation of an IBM Cloud account and their use does not generate any costs. A comparison of the noisy and exact simulations can give a general estimate of the expected fidelity with which a quantum circuit can be executed.

15. To import an existing fake backend, check the list of devices for which this is available. To do this, check the backends available in the *qiskit.providers.fake_provider* module of Qiskit.
16. Run the following code snippet which adds the noise profile of this backend and simulates a state transition.

***Note:*** We chose as an example to import the *FakeToronto* backend, mimicking the noise profile of the 27-qubit IBMQ Toronto processor.

**from** qiskit.providers.fake_provider **import** FakeToronto

rulestxt = "**./networks/Giacomantonio2010.txt**"

backend = FakeToronto()CorticalNet_noisySimulatorTransition = quantumStateTransition(rulestxt, Tmax=1, nrshots=1000, backend=backend, optimization_level=3)

print(CorticalNet_noisySimulatorTransition)

***Note:*** Since there is a limited set of quantum gates available on the IBMQ Toronto device, the operations performed in the circuit are first transpiled, i.e. decomposed, into these native gates. This transpilation also respects the coupling map of the processor, which specifies which pairs of qubits can execute 2-qubit gates. For example, to perform operations on pairs of qubits that are not connected on the coupling map, swap gates are added. Therefore, the transpiled circuit will be deeper than the circuit executed on the noiseless simulator. The required time for adding the noise profile will increase significantly if more than one noisy state transition is simulated due to the increasing depth of the circuit after transpilation.

***Note:*** The parameter *optimization_level* can take integer values from 0 to 3, with 0 indicating no optimization while 3 denotes the maximal optimization to result in a decomposition that is as shallow as possible. The chosen optimized transpilation is a partially stochastic process which will yield circuits of varying depths. This can be prevented by adding an optional value for the *seed_transpiler* argument. This can be an arbitrary integer, e.g. seed_transpiler = 123, however the same value should be kept for all future simulations to ensure reproducibility. The noise profile thus applies coherence times as well as gate errors stored in the *FakeToronto()* backend model. The provided Jupyter notebook also includes a visualization which compares the resulting distributions as bar plots. Additionally, we calculate fidelity measures to quantify their similarity.

### Using parameterized circuits to gradually vary initial gene activities

**Timing: seconds**

We use rotation gates to modify the bias of the initial qubit states, thus changing the probabilities associated with attractors.

***Note:*** Using the additional arguments ‘initActivities’ and ‘thetatype’ for the quantumStateTransition() function, we can continuously tune the activity of genes in the initial superposition state.

Here, initActivities is an array of n elements, with the *i-th* element indicating the bias for the *i-th* gene in the network file. These angles correspond to the polar angle on the Bloch sphere, i.e., 0° is identical to the default ‘0’ state and a $R_{y}\left(\theta =90{}^{\circ}\right)$ rotation would yield the same result as the previously used Hadamard gates. The argument ‘thetatype’ specifies whether the rotation is given as an ‘angle’ or ‘radian’.

17. Call the function in the following code snippet, which exemplarily simulates a steady increase in the initial activity of the first component listed in the network, Fgf8, please also refer to Weidner et al.^1^ (supplement section 4).
18. Verify that for θ=0°, only the 10010 attractor is found, indicating that the entire subspace of $2^{n-1}=16$ states where Fgf8 is inactive leads to this attractor. The weight of the 01101 should then gradually increases with the bias of the tuned component towards the ‘1’ state.

*#Variation in first gene Fgf8*

thetavalues = list(range(0,190,10)) #vary angle from 0° to 180°

**for** theta **in** thetavalues:

 print("**Gate used for initialising the first gene is R_y(theta = "** + str(theta) + "°)")

 ThetaVariationGiacomantonio = quantumStateTransition(rulestxt, Tmax=4, nrshots=1000, initActivities=[theta,90,90,90,90], thetatype="angle")

 print(ThetaVariationGiacomantonio)

### Simultaneous knockout and overexpression simulations by use of superposition perturbations

**Timing: seconds**

In this section, we use superposition states as perturbations of the network dynamics. This is done by using the initActivities and initPerturbed arguments of the quantumStateTransition() function.

***Note:*** ‘initActivities’ specifies if genes should be perturbed with a particular superposition state. We do this using an additional Boolean vector: initPerturbed. If entries in this vector are True, the corresponding initial state created by the $R_{y}\left(\theta \right)$ rotations specified in initActivities will be re-used after every quantum state transition.

***Note:*** For angles that are not 0° or 180°, this is equivalent to simultaneously performing a classical knockout (KO) and overexpression (OE) simulation, both of which may alter the system’s attractor landscape.

19. Execute the first function call in the following code snippet where the third network component Pax6 is perturbed with a bias towards OE.

***Note:*** This returns attractors from both a KO and OE simulation, however those of the OE perturbation will contribute with larger probabilities than would be the case in an unbiased averaging of attractor basin sizes.

20. Execute the second function call, where a second perturbation with a bias towards KO for the fifth component, Coup_tfi, is added. This returns the union set of all attractors from the four possible double perturbations.

SingleSuperpositionPerturbation = quantumStateTransition(rulestxt,

Tmax=4, nrshots=1000, initActivities=[90,90,135,90,90],

initPerturbed=[**False, False, True, False, False**], thetatype="**angle**")

print("**Single superposition perturbation of Pax6, biased towards OE**:")

print(SingleSuperpositionPerturbation)

print("**Double superposition perturbation of Pax6 and Coup_tfi, biased towards OE and KO respectively:**")

DoubleSuperpositionPerturbation = quantumStateTransition(rulestxt,

Tmax=4, nrshots=1000, initActivities=[90,90,135,90,45],

initPerturbed=[**False, False, True, False, True**], thetatype="**angle**")

print(DoubleSuperpositionPerturbation)

### Performing inverted state transitions by amplitude amplification

**Timing: seconds**

We exploit the reversibility of quantum circuits in combination with Grover’s algorithm to amplify the probability of states that are predecessors of a specified attractor.

***Note:*** The following code generates a circuit implementing G=2 iterations of the Grover operator, i.e. two repetitions of oracle and diffuser. The marked state is the attractor 01101, whose predecessors and pre-predecessors (nrTransitions = 2) will be amplified. That is, this Grover search effectively performs inverted state transitions. Single-state attractors such as 01101 are their own predecessors, thus all four states in the basin of attraction of this attractor (01101, 11101, 11001, 01001) will have their probabilities of measurement increased. Repeated runs and measurements of this circuit will yield a probability distribution over all possible $2^{5}=32$ states of this 5-gene network.By repeated measurement, we found that the circuit has a probability of 93.4% to yield one of these four states in the basin of attraction even though they make up only 12.5% of the system’s state space.

To reproduce the same analysis.

21. Run the following code snippet to amplify the predecessor states of the ‘markedState’ attractor 01101 using G = 2 iterations of the Grover operator.
22. Verify that the solution probability is larger than the 4/32 = 12.5% probability which would be expected from random sampling.

***Optional:*** Change the parameter G to see how the amplification of the four solution states varies. The degree to which predecessor states are amplified depends on the number of iterations G. The optimal value for this parameter depends on the total number of solutions, which can be determined by quantum counting, as described further below.

print("**G=2 iterations:**")

InvertedTransitionsCircuit_G2 =

generate_groverSTGinversion_circuit(rulestxt, nrTransitions=2,

markedState=[0,1,1,0,1], G=2)

result_G2 = execute(InvertedTransitionsCircuit_G2,

backend=Aer.get_backend('**qasm_simulator**'), shots=1000).result()

countDict_G2 =

outputformat(result_G2.get_counts(InvertedTransitionsCircuit_G2),

normalizeOutput=**True**, sortOutput=**True**, digits=3)

print(countDict_G2)

solution_prob_G2 = countDict_G2[ "11001"] + countDict_G2["01001"] +

countDict_G2["01101"] + countDict_G2["11101"]

print("**After G=2 iterations, the M/N=4/32 solution states have a**

**cumulative probability of** " + str(solution_prob_G2) + ".")

### Basin size estimation using quantum counting

**Timing: seconds to minutes**

In this section, we use a quantum counting algorithm to count the total number of solutions states instead of amplifying their probabilities as in the previous section.

***Note:*** The quantum counting algorithm combines the Grover operator with a quantum Fourier transform to estimate the total number of solutions M of a Grover search. Results are measured on a separate readout register of qubits, whose length ‘r_registerLen’ determines the accuracy of the obtained value. The measured bitstrings here no longer correspond to gene activity but instead represent the binary encoding of an integer value for M. In the examples shown below, the state 00000 has 14 immediate predecessors, while the attractor 01101 has a basin size of 4, which is reached after at least two inverted transitions, as in the Grover search before.

23. Run the first function call to QuantumCountingAlgo() in following code snippet, which estimates the number of immediate predecessors of the state 00000 (14 states in total in the classical system).
24. Run the second function call to QuantumCountingAlgo(), which estimates all predecessors of the 01101 attractor up to 2 transitions away from the attractor (4 states in total in the classical system).

CountingResults_00000_Tinv1 = QuantumCountingAlgo(rulestxt,

nrTransitions=1, markedState=[0,0,0,0,0], r_registerLen=5, nrshots = 1000)

print(CountingResults_00000_Tinv1)

CountingResults_01101_Tinv2 = QuantumCountingAlgo(rulestxt,

nrTransitions=2, markedState=[0,1,1,0,1], r_registerLen=5, nrshots =

1000)

print(CountingResults_01101_Tinv2)

### Submitting tasks to IBM’s quantum processors

**Timing: seconds to hours, depends on queue and whether the device is currently online or undergoing maintenance**

In this section we submit the circuits we created to the IBM quantum devices to be run.

***Note:*** IBM Qiskit Runtime is a new framework used to access IBM’s quantum processing units, offering in-built optimization procedures aimed at simplifying commonly performed analyses. A prominent feature of the Runtime framework is the possibility to apply some techniques of error suppression and mitigation in an automated way.

Making use of the code in the snippet below, execute the following steps.

25. Define a runtime service based on the account previously saved (in the snippet).
26. Generate a circuit for a single state transition from a uniform superposition state (in the snippet).
27. Transpile the generated circuit to match the set of gate operations available on the ‘ibm_algiers’ quantum device (in the snippet).
28. Monitor the status of the running job on the quantum device, after it gets assigned a job_id, under ‘Jobs’ in the associated instance in the IBM quantum cloud (on the IBM cloud webpage). See troubleshooting problem 1, problem 2, problem 3.
29. Read the results from the dictionary saved in the “result” variable.

***Note:*** The ‘binary_probabilities()’ line indicates that states are to be listed as bitstrings as before instead of decimal integers (e.g. without this line the result would be presented in such a way that 00011 --> 3), to more easily evaluate which network components are active or inactive.

**from qiskit_ibm_runtime import QiskitRuntimeService, Sampler, Session,**

**Options**

**from qiskit import transpile**

**from qiskit.tools.visualization import plot_histogram**

*# Save an IBM Cloud account on disk and give it a name.*

service = QiskitRuntimeService(name="**StarProtocol**")

options = Options()

# The following two settings are optional

options.resilience_level = 0 #Range from 0 to 3, 1 by default

options.optimization_level = 0 #Range from 0 to 3, 3 by default

CorticalNet1Transition = quantumStateTransition(rulestxt, Tmax=1,

returnCircuitOnly=**True**)

backend = service.get_backend('**ibm_algiers'**)

CorticalNet1Transition_tr = transpile(CorticalNet1Transition, backend)

**with** Session(service=service, backend = backend):

 sampler = Sampler( options = options)

 job = sampler.run( circuits=CorticalNet1Transition_tr, shots=100)

 result = job.result().quasi_dists[ 0].binary_probabilities()

print(result)

***Optional:*** by adding the two lines of code “options.resilience_level = *num*” and “options.optimization_level = *num*” it is possible to access and modify the error mitigation and error suppression techniques currently available within the Runtime framework. Both parameters can range from 0 (no mitigation/suppression) to 3 (maximum mitigation/suppression).

Further explanations on how these methods work can be found online at the Qiskit webpage (Mitigation: https://qiskit.org/documentation/partners/qiskit_ibm_runtime/how_to/error-mitigation.html ,

Suppression: https://qiskit.org/documentation/partners/qiskit_ibm_runtime/how_to/error-suppression.html)

### Extra error mitigation techniques (optional)

**Timing: Minutes to hours**

In this section we make use of error mitigation techniques to improve the quality of the results provided by the quantum device.

***Note:*** On top of the error mitigation and suppression techniques offered by the runtime framework, other methods can be used to better refine the results one can obtain from a quantum computer. We provide here one example: *Multiple transpilation.* The transpilation of an algorithm on to a quantum device is not a unique process, but can be performed in different, yet theoretically equivalent ways. The performance of these different transpiled algorithms on a quantum device can be very different though, providing a motivation to try to find the optimal one to perform the required analysis. As Qiskit transpilation methods, though relying on several optimization methods, still involve stochasticity to a certain degree, it is possible to perform the transpilation of a given algorithm multiple times and select among those the one thought to be the best one upon a chosen criterion. In our case, we choose the transpilation with the least number of CNOT gates involved.

To perform Multiple transpilation:

30. Define the quantum circuit to be executed (CorticalNetTransition in the snippet below).
31. Transpile multiple times the same circuit: in general these transpilations will be different from each other.
32. Count the number of CNOT gates present in each of the different transpilations.
33. Select as the preferred choice the transpilation with the least number of CNOT gates.
34. Run the selected quantum circuit with the same method used for the circuits above. See troubleshooting problem 1, problem 2, problem 3.

service = QiskitRuntimeService(name=name) *#Login to IBMQ runtime service*

backend = service.get_backend('**ibm_algiers'**) *#Configure the quantum*

*device to be used*

CorticalNet1Transition = quantumStateTransition(rulestxt, Tmax=1,

returnCircuitOnly=**True**) *#Create the circuit simulating the Boolean*

*network to be analyzed*

trans_qc_list = transpile([CorticalNet1Transition]∗ 20, backend,

optimization_level=3) *#Generate 20 different transpilations for the*

*circuit to be run*

cx_count = [circ.count_ops()[ 'cx'] **for** circ **in** trans_qc_list] *#Count the*

*number of CNOT gates contained in each different transpilation*

best_idx = np.where(cx_count == np.min(cx_count))[0][0] #Pick the transpilation with the least amount of CNOT gatesbest_qc = trans_qc_list[best_idx] *#Set given transpilation to be the*

*circuit to run*

CorticalNet1Transition_tr =

transpile(best_qc,backend, optimization_level=0)#Create the transpiled

circuit with the features evaluated above

*#Run everything on the cloud platform*

with Session(service=service, backend = backend):

 sampler = Sampler( options = Options(resilience_level=0))

 job = sampler.run( circuits=CorticalNet1Transition_tr, shots=100)

 result = job.result().quasi_dists[0].binary_probabilities()

print(result)

plot_histogram(result)

### Running quantum circuits on an IonQ’s QPU using AWS braket

**Timing: hours, depending on time at which task was sent and queue of the device**

In this section, we send a task for the simulation of a quantum state transition to the Amazon Braket cloud using the account that was set up in the “Before you begin” section.

To run tasks on the IonQ quantum processor, provide the name of your bucket and a folder inside that bucket after having saved your keys using “aws configure”.

***Note:*** The estimated cost for all tasks run using your AWS account can be seen by clicking your account name in the top right corner --> “Account” --> “Bills”. Additional payment methods can be added under “Account” --> “Payment preferences”. When a new credit card is added, AWS will send a 1,00$ authorization charge to verify the legitimacy of the card.

35. Replace the variables “my_bucket” and “my_prefix” with your specific information as described in the “before you begin” section.
36. Run the code snippet to obtain a cost estimate.

**from** braket.aws **import** AwsDevice

nrshots = 100

cost = 0.3+0.01∗nrshots #$0.30 / task + $0.01 / shot

print("**NOTE: Running a task on the IonQ backend with this number of shots**

**will create costs of $" + '{0:.2f}**'.format(cost) + " **USD!**")

*#Specify your account specific data here*

my_bucket = "amazon-braket-YOUR-BUCKET-HERE"

my_prefix = "YOUR-FOLDER-NAME-HERE"

s3_folder = (my_bucket, my_prefix)

device = AwsDevice( "arn:aws:braket:us-east-1::device/qpu/ionq/Harmony")

*#real IonQ QPU, larger device named Aria is also available*

*#device = AwsDevice("arn:aws:braket:::device/quantum-*

*simulator/amazon/sv1") #SV1 state vector simulator, costs $0.075 USD per*

*minute of run time*

***Note:*** If the QPU is offline, we can use the SV1 simulator for testing purposes.

For more information on this simulator, go to Devices--> SV1 in the Amazon Braket Dashboard or use the following link: https://docs.aws.amazon.com/braket/latest/developerguide/braket-devices.html#braket-simulator-sv1.

37. Run the following code, which generates a circuit performing a single state transition, analogous to the one shown in the previous analysis using Qiskit Runtime, sends out the task and prints the results. See troubleshooting problem 1, problem 4, problem 5, problem 6.

GiacoCirc = synthesizeFullNetworkUpdateCircuit(rulestxt,

update="**synchronous**")

n= 5 InitCircuit = QuantumCircuit(2∗n, n)

**for** q **in** range(5):

InitCircuit.h(q)

GiacoCirc = InitCircuit.compose(GiacoCirc, list(range(0, 2 ∗ n)),

list(range(0, n)))

IonQCircuit = QiskitQASM2IonQCompiler(GiacoCirc,

gatenameMapping=gatenameMapping_Qiskit_IonQ)

print("**Circuit depth:**")

print(IonQCircuit.depth) #Depth of 140 for single Giacomantonio

transition circuit

now = datetime.now()

current_time = now.strftime( "**%H:%M:%S**")

print("**Current Time =**", current_time)

task = device.run(IonQCircuit, s3_folder, shots=nrshots,

poll_timeout_seconds=24∗60∗60∗20) #20 day polling time before task times

out

resultCounts = task.result().measurement_counts

print(resultCounts)

now = datetime.now()

current_time = now.strftime( "**%H:%M:%S**")

print("**Current Time =**", current_time)

#IonQ measures all qubits -> Parse output back into same format as was

used in Qiskit simulations

qubitindices = [5,6,7,8,9] # Which qubits carry the outputs at t=1 for

nodes in order, 0-indexed

resultCounts_qiskitFormat = ionqDict2qiskitNotation(resultCounts,

qubitindices=qubitindices, invertEndian=**True**)

print(resultCounts_qiskitFormat)

***Note:*** To run circuits generated in Qiskit on the IonQ processor, we rewrite the circuits to be executable on the natively available set of basis gates of the device. The returned results are also parsed back into dictionaries in the qiskit format. This is necessary as gate names differ between the two frameworks. For the IonQ device, all qubits are measured. The probabilities concerning only the activities of the desired qubits corresponding to the output of the state transition are then summed up. These transformations are performed by the custom functions QiskitQASM2IonQcompiler() and ionqDict2qiskitNotation().

***Note:*** The *poll_timeout_seconds* argument when running a circuit using Braket gives the value in seconds for which a task will be polled for results. It is set to a default value corresponding to 5 days. Depending on the current queue, shorter values may lead to a timeout before the task is finished.

The average expected queue time can be checked at https://status.ionq.co/ along with the current status of the QPU. This site also lists any recent incidents which may be informative if a job is not being executed.

## Expected outcomes

If the simulations performed by the code snippets in this protocol have been executed correctly, the results from both real quantum processors as well as local simulators should be returned in the form of dictionaries. These dictionaries contain the results of repeated ‘shots’ of quantum circuits, meaning the repeated processing of initializing, running and measuring the circuit, yielding a single specific bitstring each time. The keys of these dictionaries encode gene activities, read from right to left, i.e., “00001” indicates that the gene listed first in the network’s text file is active. The values indicate probabilities of obtaining this state as a measurement outcome. For tasks sent to the cloud, these jobs can be monitored under the respective “Jobs” or “Tasks” sections in the IBM Quantum and Braket dashboards.

## Limitations

The execution of this protocol is dependent on the uptime of the quantum processing units used to execute the quantum circuits. These devices undergo regular maintenance. During this time, or if the device is offline for any other reason, previously sent tasks will remain in the queue and need not to be sent out again.

Furthermore, the time point of the latest calibration of the device may have an effect on the results, which can not be controlled by the user.

For the IonQ device, uptime follows a schedule of being online from 13:00 to 2:00 UTC on weekdays.

IBM devices may be offline or have a paused queue without previous notice. The state of these devices can be checked on the IBM Quantum cloud dashboard on https://cloud.ibm.com/quantum/resources/systems under “Your resources”.

The quantum cloud services of both IBM and Amazon are continuously evolving and being updated.In case of future changes, the official documentation may provide further information: https://quantum-computing.ibm.com/docs/ https://docs.aws.amazon.com/braket/index.html Additionally, the user may check the GitHub repository listed in the data and code availability statement for future updates.

## Troubleshooting

### Problem 1

Tasks for the real quantum processors are stuck in a queue and are not being executed (steps 28,34,37).

### Potential solution

Check the status of the processors for notifications informing about potential issues with the hardware preventing execution.

In the IBM Quantum cloud, the status of a backend can be checked under ‘Compute resources’ --> ’Your resources’. For AWS Braket such information can be found under ‘Devices’ --> ’IonQ’ or directly under ‘Announcements’. Alternatively, the status of IonQ devices can be monitored at https://status.ionq.co/.

### Problem 2

Tasks on the IBM quantum cloud are running but are being canceled before the task finishes (steps 28, 34).

### Potential solution

Check if the current task has surpassed the cost limit of the instance and if so, increase the limit. Note that the cost limits refer to the total runtime of all tasks that have so far been run on the instance and not any remaining runtime to be added.

### Problem 3

Tasks on the IBM quantum cloud are returning a “401 Client Error: Unauthorized for url …” (steps 28,34).

### Potential solution

Check that the QiskitRuntimeService() function call uses the “ibm_cloud” channel and that both the API token and CRN are correct. If the error persists, create a new API token under https://cloud.ibm.com/iam/apikeys.

### Problem 4

Tasks for the IonQ device do not execute, giving an error of the type “Could not connect to the endpoint URL” (step 37).

### Potential solution

Select a different region, e.g., “us-east-1” for the IonQ device, as QPUs are region specific. Rerun the command “aws configure” and update this information. Create a new bucket in the new region.

### Problem 5

Tasks for the IonQ device do not execute, giving an error of the type “InvalidSignatureException when calling GetDevice operation: Signature expired…” (step 37).

### Potential solution

Synchronize your device’s clock. Under Windows this can be done via “Settings” --> ”Date and time settings” --> “Sync now”.

### Problem 6

Tasks for the IonQ device do not execute, giving an error of the type “An error occurred (AccessDeniedException) when calling the GetDevice operation: User … is not authorized to perform: braket:GetDevice on resource: … /qpu/ionq/Harmony” (step 37).

### Potential solution

Open the IAM dashboard, go to “Access Management”--> “Users” and select your username. Go to “Add permissions” and add the permission policy “AmazonBraketFullAccess” to this user.

## Resource availability

### Lead contact

Further information and requests for resources and reagents should be directed to and will be fulfilled by the lead contact, Hans A. Kestler (hans.kestler@uni-ulm.de).

### Materials availability

This study did not generate new unique reagents.

## Data Availability

The code generated during this study is available at https://github.com/sysbio-bioinf/QuantumSTARProtocol or alternatively via Zenodo under https://doi.org/10.5281/zenodo.8023318. This includes the rules of the Boolean networks used in this protocol as well as the code required for running the presented analyses as a Jupyter notebook. Please note that libraries and functions related to Qiskit are subject to frequent updates and changes and may become deprecated in the future.
